# The Influence of Worry and Nervous Shock on the Uterus and Its Functions

**Published:** 1884-07

**Authors:** 


### Selections.

### THE INFLUENCE OF WORRY AND NERVOUS SHOCK ON THE UTERUS AND ITS FUNCTIONS.

Clinical lecture by Wm. Goodell :

This lady is 37 years old. She has had four children, the youngest of which is nine years old. Her trouble began twelve years ago, but has been worse since the birth of her last child. She complains of pain in the back and head, fluttering of the heart, cold hands and feet, irritable bladder, wakefulness, and when she does sleep has bad dreams, which wake her in a fright.

What is the meaning of these symptoms? They are nervous symptoms. We must decide whether they are due to a local or constitutional disease with a constitutional expression, or whether to a pure nervous trouble. In the first place, I ask her whether she has had any trouble in her family. She tells me that she has had no trouble; she has had a happy home and no cause of worry.

I have a patient who often comes to see me, complaining with these nervous symptoms, and I have repeatedly told her that she must have had some cause of worry or of nervous shock, but she has always denied any such cause until a day or two ago, when she said that possibly a passage in her history might explain the trouble. She said that her father had softening of the brain and became insane, but the insanity was of a passive type. He had never done any one harm until one day when she entered his room, he made a spring at her, and she saw from his expression that he was going to do her harm. She seized some heavy article, exclaiming, "Father, if you come near me, I shall throw this at you," and looking him steadily in the eye, he quailed. In order to get out of the door, she had to turn her back towards him, and the instant she did this, he sprang and caught her and tried to bite and injure her. She shrieked for assistance, which fortunately was at hand. This fright made such an impression upon the nerve-centers that from that time she was never well. This was the foundation of her troubles, although she thought that it had nothing to do with them.

I have had a patient under my care for seven months, suffering from menorrhagia and these nervous symptoms, in whom the trouble was caused by the throwing of a dead cat into her lap. You may ask, how can a dead cat produce menorrhagia? or, in other words, how can a fright produce menorrhagia? It does in this way: It produces a profound impression upon the nerve-centers, and there is no longer that correlation of nervepower which exists in health. The nerves behave as they please. There is irregular distribution of nerve fluid; this leads to irregular distribution of blood; more blood goes to one organ than to another. The most exacting organs during the woman's menstrual life are the womb and its annexes, the ovary, the vagina, the erectile tissue around the womb, the broad ligaments and the fallopian tube. They receive more blood than they should. There is increased nutritive action, vegetations develop, and the womb increases in size. In the lady of whom I have spoken, I was unable to cure the menorrhagia. I used the curette and could control the hemorrhage for a time, but it would always return, and ultimately I removed both ovaries. These organs were found to have undergone follicular degeneration.

I shall now make a vaginal examination. I examined her once before, but did not think that any operation was required. There has been a laceration of the cervix and of the perinæum, but these lesions do not usually produce such severe symptoms. She has worn a pessary, but it always caused pain. I must honestly say that I dislike to subject this woman to an operation when I am by no means positive that it will do her good, but I shall do something which I think will modify and tone up the condition of the womb. The sound gives a measurement of three inches. There is no ectropion of the mucous lining of the cervical canal, and that is one reason why I think an operation is not indicated. To-day I shall curette the womb.

Is there any danger in the use of the dull curette? This is an important question, for you will frequently do the operation in your office. I have never lost a patient from the use of the curette, but I have occasionally had some inflammatory disturbance.

There has been a little bleeding following the passage of the sound. I shall first introduce the blunt curette. I remove several vegetations. A few of these are quite large. These little fungous growths are entirely benign. I have often told you how unreliable the microscopical examination of uterine growths is. The best microscopists have told me that certain growths were epithelioma, when the subsequent course of the case has proven that the disease was perfectly benign. I lately came across a passage in the works of a German writer in which he says that it is one of the most difficult things to decide between benign and malignant growths of the womb by the microscope. The alveoli of innocent growths simulate very closely cylindrical epithelioma. I think that this is the reason that microscopists have been misled.

Having removed so many of these vegetations, I shall go over the endometrium with the sharp curette. This is an instrument I do not advise you to use at first, for your fingers are all thumbs, and you cannot manipulate with the delicacy that is necessary in employing this instrument. I first dilate the canal with Ellinger's dilator, in order that the instrument may readily enter, and then, taking it as I would a pen, I gently pass it into the cavity of the womb. I can hear and feel the curette passing over the rough vegetations. The lining of the womb should be perfectly smooth. Having removed all the vegetations, I shall make an application of strong tincture of iodine to the fundus. She will then be put to bed, and, if necessary, receive an opium suppository.

What shall we give this patient internally? If she were rich enough I should put her to bed, have her well rubbed, and electricity applied. She cannot do this. The next best thing is to give some remedy to tone down these nerve-centers which are not behaving themselves. I should like to give her the valerianate of zinc and quinia, which, take it all in all, is the best combination which we can give. I shall order a triple valerianate, as follows: ℞. Zinci valerianatis, quiniæ valerianatis, ferri valerianatis, āā gr. xx. M. et ft. pil., No. xx. Sig. One, three times a day.

After a time, I shall give strychnia in small doses, gradually increased. She would, I think, be benefited by the use of malt. Keasbey and Mattison make a good preparation. Of this, she will take a tablespoonful three times a day in milk. It is a good plan to take one dose of the malt at bed-time. This favors sleep. (The patient was now removed.)

I tell you frankly that I do not believe her statement that she has had no trouble; I believe that she has some cause of worry which has aggravated this, for there is not sufficent local trouble to account for the constitutional symptoms.

To illustrate this, let me refer to a case which I saw the other day. A young girl was sent to me with the history of dysmenorrhœa, and all the train of nervous symptoms which the patient before you to-day exhibited. I examined her, and, as a matter of course, found anteflexion, but the sound passed readily. The dysmenorrhœa had developed within a few months. I asked her if she had studied hard at school. She said that she had, and had been compelled to leave school. She stated that she had no cause of worry. After she had left the room, the lady who came with her told me that the girl's father, although a good man, was an intemperate one, and that his intemperate habits worried the girl very much. In the great majority of such cases a cause for worriment will be found.—*Medical and Surgical Reporter.*

